# Polymorphism in the *Vesicular Monoamine Transporter 2* Gene Decreases the Risk of Parkinson's Disease in Han Chinese Men

**DOI:** 10.1155/2015/903164

**Published:** 2015-07-12

**Authors:** Xinglong Yang, Pingrong Xu, Quanzhen Zhao, Ran An, Hua Jia, Zhuolin Liu, Yanming Xu

**Affiliations:** ^1^Department of Neurology, West China Hospital, Sichuan University, 37 Guo Xue Xiang, Chengdu, Sichuan 610041, China; ^2^Department of Pharmacy, West China Hospital, Sichuan University, 37 Guo Xue Xiang, Chengdu, Sichuan 610041, China; ^3^Department of Neurology, First Affiliated Hospital of Sun Yat-sen University, Guangzhou, Guangdong 510080, China

## Abstract

*Background.* Polymorphisms rs363371 and rs363324 in the* vesicular monoamine transporter 2* (*VMAT2*) gene have been associated with risk of PD in an Italian population, and our aim is to investigate the association between the two single-nucleotide polymorphisms and PD in Han Chinese.* Methods.* 561 Han Chinese PD patients and 491 healthy age- and gender-matched controls were genotyped using Ligase detection reaction (LDR) method.* Result.* Both of patient and control groups showed similar genotype frequencies between patients and controls at both rs363371 and rs363324, as well as similar minor A allele frequencies at rs363371 (*P* = 0.452) and rs363324 (*P* = 0.413). None of the observed haplotypes showed a significant association with PD. Subgroup analysis by gender and age at onset revealed a significant association between the A allele of rs363371 and PD in Han Chinese males relative to healthy controls (OR 0.799, 95%  CI 0.665 to 0.959, *P* = 0.016), and this association remained significant after adjusting for age (OR 0.785, 95%  CI 0.652 to 0.945, *P* = 0.011).* Conclusion.* These results suggest that polymorphism of* VMAT2* locus is associated with risk of PD in Han Chinese overall but that the A allele at rs363371 may protect against PD in Han Chinese males.

## 1. Introduction

Parkinson's disease (PD) is a neurodegenerative disorder characterized by static tremor, bradykinesia, rigidity, posture, gait dysfunction, and several nonmotor symptoms. The main pathological features of PD are aggregation of *α*-synuclein and selective loss of dopaminergic neurons in the substantia nigra pars compacta. Both motor and nonmotor PD symptoms are thought to be associated with protein and lipid oxidation mediated by the monoamine neurotransmitters dopamine and norepinephrine; this oxidation damages dopaminergic cells, as shown in animal studies of PD induced by 6-hydroxydopamine (6-OHDA) [1, 2]. Taken together, previous studies indicate that the storage and metabolism of monoamines, particularly dopamine, can influence levels of oxidative stress, which can in turn lead to PD.

Oxidative stress has been shown to arise from cytoplasmic dopamine rather than extracellular dopamine [3]. Homeostasis of cytoplasmic dopamine depends on normal functioning of both the dopamine transporter (DAT), which recycles dopamine from the extracellular medium into the cytoplasm, and the vesicular monoamine transporter 2 (VMAT2), which packages dopamine into vesicles and helps transport it to the extracellular medium [4]. VMAT2 dysfunction leads to excessive dopamine aggregation in the cytoplasm, where its metabolism generates reactive oxidative species such as ^∙^OH, O_2_^∙−^, H_2_O_2_, and neurotoxic quinones [2, 5, 6]. Thus VMAT2 dysfunction may contribute to PD via two pathways. One is that the reactive oxidative species inhibit mitochondrial respiration [7, 8], proteasome activity [9], and intracellular antioxidative defenses [10, 11], directly causing the degeneration of dopaminergic neurons in substantia nigra. The second pathway is through interaction between VMAT2 and *α*-synuclein. The *α*-synuclein protein is thought to contribute to PD pathogenesis by disturbing ion homeostasis, mitochondrial function, and proteostasis, as well as by inducing neuroinflammation [12]. VMAT2 hypomorphic mice overexpress *α*-synuclein in nigral dopamine neurons and show significantly more dopaminergic neurodegeneration than wild-type mice. This suggests that VMAT2 dysfunction leads to elevated intracellular dopamine levels, which may render dopaminergic neurons more susceptible to *α*-synuclein-mediated degeneration [13]. Thus, VMAT2 function may be essential to prevent endogenous dopamine from becoming neurotoxic [14].

It is not surprising, then, that two single-nucleotide polymorphisms (SNPs) in the promoter region of the* VMAT2* gene, rs363371 and rs363324, have been associated with risk of PD in an Italian population: the A allele of rs363371 and the G allele of rs363324 were reported to decrease the risk of developing PD [15]. Studies of PD risk and other* VMAT2* SNPs have come to different conclusions: two studies in Japan showed a negative association [16, 17], while four genome-wide association studies in Caucasians showed no association [18–21]. To verify whether rs363371 and rs363324 are associated with PD risk in other ethnic groups, the present study compared Han Chinese patients and controls. These findings complement the known association of SNPs in the dopamine transporter gene with risk of disease [22–24].

## 2. Subjects and Methods

### 2.1. Subjects

A total of 561 Han Chinese patients with sporadic PD and 491 unrelated, healthy Han Chinese control subjects were consecutively recruited from the movement disorder centers of West China Hospital, Sichuan University, located in southwest China, and of the First Affiliated Hospital of Sun Yat-sen University, located in southeast China. Clinical diagnosis of PD was established by two independent movement disorder specialists according to UK PD Society Brain Bank criteria for idiopathic PD [25]. Patients with at least one relative with PD were excluded. A neurologist confirmed that controls were free of any neurodegenerative disorders and family history of neurodegenerative disease for at least one generation. Written informed consent was obtained from all individuals involved in this research, and the study protocol was approved by the Ethics Committees at West China Hospital of Sichuan University and at the First Affiliated Hospital of Sun Yat-sen University.

### 2.2. Genotyping

Genomic DNA was obtained from peripheral leukocytes using classical phenol-chloroform extraction. All genotyping was performed by Shanghai Biowing Applied Biotechnology (Shanghai, China) using ligase detection reactions [26]. Target DNA sequences in the* VMAT2* gene were amplified using a multiplex PCR method and the following sequences: rs262271 forward, 5′-GATGAACCCAAGGCAGGAAC-3′; rs363371 reverse, 5′-CTCACATGGCACAATGAATG-3′; rs363324 forward, 5′-CCCTGGAACTAATTCCTGTC-3′; rs363324 reverse, 5′-AAATGCCGATGGACCAGTTC-3′. After amplification, 1 *μ*L of proteinase K (20 mg/mL) was added and the reactions were heated at 70°C for 10 min and then quenched at 94°C for 15 min. The ligation reaction for each subject was carried out in a final volume of 20 *μ*L containing 20 mM Tris-HCl (pH 7.6), 25 mM potassium acetate, 10 mM magnesium acetate, 10 mM DTT, 1 mM NAD, 0.1% Triton X-100, 10 *μ*L of multiplex PCR product, 1 pmol of each discriminating oligo, 1 pmol of each common oligo, and 0.5 *μ*L of 40 U/*μ*L Taq DNA ligase (New England Biolabs, USA). The ligase detection reaction was then performed using 40 cycles of 94°C for 30 s and 63°C for 4 min. Fluorescent products were fractionated and analyzed using an ABI sequencer 377.

The technicians performing the genotyping were blinded to the case or control status of the samples. For quality control, 20% of the samples were randomly selected and processed independently by another researcher; all samples gave identical results in both researchers' hands. We also selected 10 samples for each genotype of the two SNPs obtained by ligase detection reaction, and for more comprehensive characterization, we sequenced around the two SNPs using an ABI Prism 3730 automated sequencer, which showed 100% reproducibility.

### 2.3. Statistical Analysis

Age for different groups was reported as mean ± standard deviation (SD), while gender, allele, and genotype were reported as percentages. Allele and genotype frequencies were determined by direct counting of alleles. Genotype distributions were tested for concordance with Hardy-Weinberg equilibrium (HWE). Differences in gender and age between PD and control groups were assessed for significance using, respectively, the chi-squared test and *t*-test. Logistic regression analysis was performed using PD as the dependent variable, while age, gender, and genotype (allele) of the two SNPs served as independent variables. All statistical tests were carried out using SPSS 17.0 (IBM, Chicago, USA), using a significance threshold of *P* < 0.025 (corrected for multiple comparisons). Haplotype analysis was performed using SHEsis 4.0 online (http://analysis.bio-x.cn/myAnalysis.php). Genotype imputation was performed using IPUTE2 (http://mathgen.stats.ox.ac.uk/impute/impute_v2.html).

## 3. Results

### 3.1. Basic Characteristics of PD and Control Subjects

A total of 561 Han Chinese patients with PD (62.73 ± 11.74 years) and 491 healthy controls (61.8 ± 8.48 years) were included in the study. The PD and control groups were well matched by age and sex (Table 1). Among the PD patients, 167 had early-onset PD (EOPD), defined as disease with onset at 50 years; mean age at onset in this group was 44.55 ± 4.90 years. The remaining 394 had late-onset PD (LOPD), and the mean age at onset was 63.76 ± 6.64 years.

### 3.2. Comparison of Genotype and Allele Frequencies between PD Patients and Controls

Genotype distributions at rs363371 were in HWE in patients (*P* = 0.597) and controls (*P* = 0.228); the same was true at rs363324 for patients (*P* = 0.116) and controls (*P* = 0.592). Genotype frequencies at both loci were similar between patients and controls (*P* > 0.05), and frequencies of the A allele were similar between both groups at both rs363371 (*P* > 0.05) and rs363324 (*P* > 0.321) (Table 2).

Next we performed subgroup analysis based on gender and genotype. Allele frequencies at rs363324 were similar between patients and controls in all subgroup analyses (Table 4). In contrast, we identified a significant difference in the frequency of the A allele at rs363371 between male PD patients and the male control group (OR 0.799, 95% CI 0.665 to 0.959, *P* = 0.016). This association remained significant after adjusting for age (OR 0.785, 95% CI 0.652 to 0.945, *P* = 0.011). No other subgroup analyses showed significant differences between patients and controls (Table 3).

### 3.3. Haplotype Analysis

The linkage disequilibrium *D*′ value was 0.343 for rs363371 and rs363324, suggesting a strong recombination event. Haplotype analysis, in which haplotypes with frequencies <0.03 were excluded, identified the three haplotypes A-G, G-A, and G-G. None of these haplotypes showed a significant association with PD (Table 5).

### 3.4. Imputation

To provide independent verification of our findings, we used IMPUTE2 [27, 28] to perform direct imputation of the chromosomal region* chr10:118980000-119041941*, which includes the entire* VMAT2* gene and an additional 5 kb on either side of the gene. We included the option* buffer*〈250〉 to force IMPUTE2 to use an internal buffer region of 250 kb on either side of the analysis window [27]. A dataset of 1000 genomes was downloaded from the IMPUTE2 website (http://mathgen.stats.ox.ac.uk/impute/impute_v2.html) [28] to serve as the imputation reference. The threshold for genotype calling was contained in the -g file (http://www.stats.ox.ac.uk/~marchini/software/gwas/file_format.html). For each SNP in each individual, the program uses the genotype with the maximum probability if that probability exceeds the threshold; otherwise, the genotype is treated as missing. An -Ne value of 20000 was used according to the manufacturer's instructions to control effective population size in the population-genetic model used by the software. Nine SNPs were obtained after imputation: chr10:118987954:D, rs10886050, rs2532798, rs2532799, rs2619102, rs2619103, rs2803820, rs3026047, and rs4081624. After checking HWE and performing logistic regression, none of the nine SNPs was found to differ clearly between the case and control groups (see Supplementary Materials 1 and 2 available online at http://dx.doi.org/10.1155/2015/903164).

## 4. Discussion

Our study, inspired by the reported association between* VMAT2* polymorphism at rs363371 and rs363324 and risk of PD in Italians [15], failed to reproduce these associations in a Han Chinese population. None of the three haplotypes identified in our population showed a significant association with disease. Even after imputation analysis, no obvious association was detected between* VMAT2* and PD. On the other hand, subgroup analysis identified the A allele of rs363371 as protecting male Han Chinese from developing PD. These findings suggest that the effects of* VMAT2* polymorphism on PD risk depend on ethnicity, highlighting the need for further study of these polymorphisms in other populations.

Our detection of an association between rs363371 and PD only in male Han Chinese contrasts with several lines of evidence implicating VMAT2 in PD pathology. A member of the SLC18A2 family of transporters, which are expressed primarily in the brain, VMAT2, is a 12-pass transmembrane protein [4] that packages dopamine and neurotoxins into secretion vesicles [29]. VMAT2 is expressed in the nigrostriatal area at 100-fold higher levels than in cerebellar or cerebral areas, and levels of nigrostriatal VMAT2 are lower in PD patients than in healthy controls [30, 31]. Studies in cell culture and animal models have provided additional evidence that VMAT2 dysfunction can lead to PD pathology. Cells overexpressing *α*-synuclein show reduced VMAT2 activity, elevated cytosolic dopamine concentrations, and elevated levels of reactive oxidative species [32, 33]. A mouse line in which nondopaminergic striatal neurons lack VMAT2 but express dopamine transporter shows motor deficits, striatal neurodegeneration, and increased dopamine oxidation [34]. A transgenic mouse line expressing only 15% of the normal amount of VMAT2 closely mimics human PD characteristics, including deficits in l-DOPA-responsive motor activity and full-scale nonmotor symptoms [35].

Drug studies are also consistent with a link between VMAT2 and PD. Systemic administration of the VMAT2 inhibitor reserpine induces PD-like motor symptoms [36, 37] and nonmotor symptoms [38] in mice, as well as oxidative stress in the striatum [39, 40] and prefrontal cortex [39] of rat brain. Rotenone is widely used to model the death of dopamine neurons in Parkinson's disease both in vitro and in vivo, and the drug works via c-Jun N-terminal protein kinase (JNK) to inhibit VMAT2 [41]. Tetrabenazine (TBZ), a reversible inhibitor of VMAT2, preferentially depletes striatal dopamine. Treating rats and mice with TBZ induces tremulous jaw movements as a model of parkinsonian tremor [42]. Treating dopaminergic cell with the VMAT2 inhibitor ketanserin exacerbates BH4-induced dopaminergic cell death and increases levels of lipid peroxidation and of protein-bound quinone, indicating greater oxidative stress [43]. Pramipexole, recommended for clinical use in early stages of PD, increases VMAT2 activity, increasing dopamine packaging and secretion in vesicles, reducing the cytosolic dopamine concentration, and thereby protecting dopaminergic neurons [44]. The antiparkinsonism drug apomorphine may work by a similar mechanism [45]. Together these studies suggest that VMAT2 is a quite promising therapeutic target against PD.

Given these numerous lines of evidence implicating VMAT2 dysfunction in PD pathology, it makes sense that we detected a protective effect of the A allele at rs363371 in Chinese males. We speculate that the protective A allele is associated with higher VMAT2 activity, and in fact this locus is in linkage disequilibrium with locus rs2619096, which doubles the activity of the* VMAT2* promoter [46]. Surprisingly, despite the evidence for a link between VMAT2 dysfunction and PD, we failed to find any association between PD and the A allele at rs363371 in Chinese females, or between PD and the other alleles and genotypes that we tested. This discrepancy from the earlier study in Italians [15] may reflect ethnic differences. For example, the frequency of the A allele at rs363371 was 54.9%–56.1% in our subjects but only 19.2%–23.4% in Italian subjects [15].

Several studies have suggested that gender can modulate PD risk, with women showing lower incidence and prevalence of the disease [47]. Some studies suggest that estrogen and its signalling pathways account for some of these differences [48, 49]. The protective effects of estrogen appear to be related to inhibiting the function of the dopamine transporter [50, 51], preventing loss of VMAT2 [52], and other pathways [53]. Thus we could speculate that estrogen exerts neuroprotective effects on nigrostriatal neurons and that this estrogen effect is much stronger than the neuroprotective effect of VMAT2. As a result, the neuroprotective effects of VMAT2 may be more obvious in men, who have a relatively low concentration of estrogen. This model is consistent with our finding that the A allele of rs363371 appears to protect Han Chinese men from developing PD.

Our findings should be interpreted with caution because we did not take into account gene-gene or gene-environment interactions. We focused on only two SNPs in the* VMAT2* gene, though we did expand this to nine in the imputation analysis, highlighting the need for more comprehensive studies of* VMAT2* polymorphism involving next-generation sequencing. Our sample size is relatively small; nevertheless, power calculations that we performed prior to data collection based on the frequencies of G allele in Han Chinese (0.444; www.ncbi.nlm.nih.gov/projects/SNP) and the OR of 0.72 in the Italian study [15] suggested an anticipated power of 0.91 for rs363371 and 0.616 for 363324. These are reasonable power values to detect clinically important associations. All our PD patients came from southern China, so the discrepancy from the Italian study of the same polymorphisms [15] may simply reflect ethnic differences. The ethnic homogeneity in our study does have the advantage of reducing the risk of allelic heterogeneity and false positive associations, while increasing tagging efficiency [54].

In conclusion, our study suggests that* VMAT2* polymorphism decreases the risk of developing PD in Han Chinese men. More studies are needed on the possible effects of* VMAT2* polymorphisms on VMAT2 activity and risk of PD in larger populations and in other ethnic groups.

## Supplementary Material

Nine SNPs were obtained after imputation: chr10:118987954:D, rs10886050, rs2532798, rs2532799, rs2619102, rs2619103, rs2803820, rs3026047, and rs4081624. After checking HWE and performing logistic regression, none of the nine SNPs was found to differ clearly between the case and control groups.

## Figures and Tables

**Table 1:** Demographic data for Han Chinese with Parkinson's disease (PD) and healthy controls.

Factor	PD (*n* = 561)	Controls (*n* = 491)	Comparison^*∗*^
Age, yr (mean ± SD)	62.6 ± 11.80	61.8 ± 8.48	*t* = 1.25; *P* = 0.21
Gender, *n*			
Male	302	289	0.80; 0.63–1.02; 3.14; 0.08
Female	259	202	

PD: Parkinson's disease; HC: healthy control.

^*∗*^Unless otherwise indicated, the values indicate OR; 95% CI; *χ* ^2^; *P* value.

**Table 2:** Distribution of genotypes and alleles at rs363371 and rs363324 among Han Chinese with Parkinson's disease (PD) and healthy controls.

	PD	Controls	OR, 95% CI, *P*	OR, 95% CI, *P* ^*∗*^
rs363371				
Genotype frequencies				
AA	166 (29.6)	148 (30.1)	—	—
AG	284 (50.6)	255 (51.9)	1.135, 0.794–1.621, 0.487	1.145, 0.798–1.643, 0.462
GG	111 (19.8)	88 (17.9)	1.147, 0.827–1.590, 0.411	1.149, 0.826–1.598, 0.409
Dominant model				
AA	166 (29.6)	148 (30.1)	—	—
AG + GG	395 (70.4)	343 (69.9)	1.027, 0.788–1.338, 0.845	1.034, 0.791–1.352, 0.804
Recessive model				
AA + AG	450 (80.2)	403 (82.1)	—	—
GG	111 (19.8)	88 (17.9)	1.142, 0.838–1.557, 0.400	1.148, 0.839–1.570, 0.389
Allele frequencies				
A	616 (54.9)	551 (56.1)	—	—
G	506 (45.1)	431 (43.9)	0.949, 0.828–1.088, 0.452	0.949, 0.827–1.090, 0.459
rs363324				
Genotype frequencies				
AA	21 (3.7)	19 (3.9)	—	—
AG	206 (36.7)	164 (33.6)	0.994, 0.524–1.885, 0.985	0.937, 0.490–1.791, 0.844
GG	334 (59.5)	304 (62.4)	0.875, 0.676–1.131, 0.308	0.865, 0.666–1.123, 0.275
Dominant model				
AA	21 (3.7)	19 (3.9)	—	—
AG + GG	540 (96.3)	468 (96.1)	1.044, 0.554–1.966, 0.894	0.989, 0.521–1.876, 0.972
Recessive model				
AA + AG	227 (40.5)	283 (74.1)	—	—
GG	334 (59.5)	304 (25.9)	0.886, 0.690–1.136, 0.340	0.872, 0.677–1.122, 0.287
Allele frequencies				
A	248 (22.1)	202 (20.7)	—	—
G	874 (77.9)	772 (79.3)	1.084, 0.893–1.317, 0.413	1.105, 0.907–1.344, 0.321

PD: Parkinson's disease; CI: confidence interval; OR: odds ratio.

^*∗*^ *P* value for binary logistic regression after adjusting for age and gender.

**Table 3:** Distribution of genotype and alleles at rs363371 in Han Chinese PD patients and controls, stratified by gender.

	PD	HC	OR, 95% CI, *P*	OR, 95% CI, *P* ^**∗**^
Males				
Genotype frequency				
AA	72 (23.8)	87 (30.1)	—	—
AG	159 (52.6)	152 (52.6)	0.835, 0.571–1.221, 0.352	0.807, 0.549–1.187, 0.276
GG	71 (23.3)	50 (17.3)	0.615, 0.383–0.989, 0.045	0.594, 0.367–0.962, 0.034
Dominant model				
AA	72 (23.8)	87 (27.4)	—	—
AA + AG	230 (76.2)	202 (72.6)	0.767, 0.535–1.101, 0.767	0.741, 0.514–1.070, 0.110
Recessive model				
AA + AG	231 (76.5)	239 (82.7)	—	—
GG	71 (23.5)	50 (17.3)	0.692, 0.462–1.037, 0.075	0.684, 0.454–1.030, 0.069
Allele				
A	303 (50.2)	326 (56.4)	—	—
G	301 (49.8)	252 (43.6)	0.799, 0.665–0.959, **0.016**	0.785, 0.652–0.945, **0.011**
Females				
Genotype frequency				
AA	94 (36.3)	61 (30.2)	—	—
AG	125 (48.3)	103 (51)	0.713, 0.472–1.079, 0.110	0.691, 0.456–1.048, 0.082
GG	40 (15.4)	38 (18.8)	0.874, 0.637–1.199, 0.405	0.871, 0.634–1.196, 0.393
Dominant model				
AA	94 (36.3)	61 (30.2)	—	—
AG + GG	165 (63.7)	141 (69.8)	1.252, 0.844–1.857, 0.264	1.290, 0.897–1.856, 0.170
Recessive model				
AA + AG	219 (84.6)	164 (81.2)	—	—
GG	40 (15.4)	38 (18.8)	1.164, 0.871–1.555, 0.305	1.185, 0.885–1.586, 0.256
Allele				
A	311 (60.4)	225 (55.7)	—	—
G	205 (39.6)	179 (44.3)	0.844, 0.685–1.035, 0.108	0.829, 0.673–1.022, 0.079

PD: Parkinson's disease; HC: healthy controls; CI: confidence interval; OR: odds ratio.

^**∗**^ *P* value for binary logistic regression after adjusting for age.

**Table 4:** Distribution of genotype and alleles at rs363324 among Han Chinese PD patients and controls, stratified by gender.

	PD	HC	OR, 95% CI, *P*	OR, 95% CI, *P* ^*∗*^
Males				
Genotype frequency				
AA	12 (4)	12 (4.2)	—	—
AG	121 (40)	98 (34.1)	0.855, 0.395–1.983, 0.766	0.914, 0.403–2.072, 0.829
GG	169 (56)	177 (61.7)	1.135, 0.513–2.510, 0.755	1.198, 0.535–2.682, 0.660
Dominant model				
AA	12 (4)	12 (4.2)	—	—
AA + AG	290 (96)	275 (95.8)	1.031, 0.466–2.279, 0.940	1.078, 0.482–2.410, 0.855
Recessive model				
AA + AG	133 (44)	110 (38.3)	—	—
GG	169 (56)	177 (61.7)	1.266, 0.957–1.676, 0.099	1.300, 0.978–1.729, 0.071
Allele				
A	145 (24)	122 (21.3)	—	—
G	459 (76)	452 (78.7)	1.181, 0.918–1.519, 0.195	1.208, 0.936–1.560, 0.147
Females				
Genotype frequency				
AA	9 (3.5)	7 (3.5)	—	—
AG	85 (32.8)	66 (33)	0.877, 0.310–2.480, 0.805	0.920, 0.323–2.619, 0.876
GG	165 (63.7)	127 (63.5)	0.880, 0.316–2.447, 0.806	0.907, 0.324–2.539, 0.907
Dominant model				
AA	9 (3.5)	7 (3.5)	—	—
AG + GG	250 (96.5)	193 (96.5)	0.879, 0.316–2.444, 0.804	0.912, 0.326–2.554, 0.861
Recessive model				
AA + AG	94 (36.3)	73 (36.5)	—	—
GG	165 (63.7)	127 (63.5)	0.991, 0.769–1.278, 0.945	0.979, 0.758–1.246, 0.868
Allele				
A	103 (19.9)	80 (20)	—	—
G	415 (80.1)	320 (80)	0.981, 0.723–1.330, 0.901	0.975, 0.718–1.325, 0.975

PD: Parkinson's disease; HC: healthy controls; CI: confidence interval; OR: odds ratio.

^*∗*^ *P* value for binary logistic regression after adjusting for age.

**Table 5:** Haplotype frequencies in Han Chinese with PD and in healthy controls.

SNP	Haplotype^*∗*^	PD, *n* (%)	Controls, *n* (%)	*χ* ^2^	*P*	OR [95% CI]
1, 2	A-G	619 (55.3)	545 (56.1)	0.135	0.713	0.968 [0.818–1.151]
	G-A	247 (22.1)	201 (20.7)	0.567	0.448	1.084 [0.880–1.337]
	G-G	254 (22.6)	226 (23.2)	0.094	0.759	0.969 [0.790–1.188]

SNP: single-nucleotide polymorphism.

1 = rs363371, 2 = rs363324.

^*∗*^Only haplotypes with a frequency of *P* > 0.03 were considered in the analysis.

A, G indicates the allele at rs363371; A, G indicates the allele at rs363324.
